# Frequent Combination of Antimicrobial Multiresistance and Extraintestinal Pathogenicity in *Escherichia coli* Isolates from Urban Rats (*Rattus norvegicus*) in Berlin, Germany

**DOI:** 10.1371/journal.pone.0050331

**Published:** 2012-11-26

**Authors:** Sebastian Guenther, Astrid Bethe, Angelika Fruth, Torsten Semmler, Rainer G. Ulrich, Lothar H. Wieler, Christa Ewers

**Affiliations:** 1 Freie Universität Berlin, Department of Veterinary Medicine, Institute of Microbiology and Epizootics, Berlin, Germany; 2 Robert Koch-Institut, Wernigerode, Germany; 3 Friedrich-Loeffler-Institut, Federal Research Institute for Animal Health, Institute for Novel and Emerging Infectious Diseases, Greifswald - Insel Riems, Germany; 4 Justus-Liebig-Universität Giessen, Department of Veterinary Medicine, Institute of Hygiene and Infectious Diseases of Animals, Giessen, Germany; University College Dublin, Ireland

## Abstract

Urban rats present a global public health concern as they are considered a reservoir and vector of zoonotic pathogens, including *Escherichia coli*. In view of the increasing emergence of antimicrobial resistant *E. coli* strains and the on-going discussion about environmental reservoirs, we intended to analyse whether urban rats might be a potential source of putatively zoonotic *E. coli* combining resistance and virulence. For that, we took fecal samples from 87 brown rats (*Rattus norvegicus*) and tested at least three *E. coli* colonies from each animal. Thirty two of these *E. coli* strains were pre-selected from a total of 211 non-duplicate isolates based on their phenotypic resistance to at least three antimicrobial classes, thus fulfilling the definition of multiresistance. As determined by multilocus sequence typing (MLST), these 32 strains belonged to 24 different sequence types (STs), indicating a high phylogenetic diversity. We identified STs, which frequently occur among extraintestinal pathogenic *E. coli* (ExPEC), such as STs 95, 131, 70, 428, and 127. Also, the detection of a number of typical virulence genes confirmed that the rats tested carried ExPEC-like strains. In particular, the finding of an Extended-spectrum beta-lactamase (ESBL)-producing strain which belongs to a highly virulent, so far mainly human- and avian-restricted ExPEC lineage (ST95), which expresses a serogroup linked with invasive strains (O18:NM:K1), and finally, which produces an ESBL-type frequently identified among human strains (CTX-M-9), pointed towards the important role, urban rats might play in the transmission of multiresistant and virulent *E. coli* strains. Indeed, using a chicken infection model, this strain showed a high *in vivo* pathogenicity. Imagining the high numbers of urban rats living worldwide, the way to the transmission of putatively zoonotic, multiresistant, and virulent strains might not be far ahead. The unforeseeable consequences of such an emerging public health threat need careful consideration in the future.

## Introduction

Brown rats (*Rattus norvegicus*) are commensal rodents found in urban areas worldwide. They are associated with hygienic problems and are considered a reservoir and vector of several zoonotic pathogens. Indeed, until the twentieth century, one of the most feared diseases related to rats was the plague caused by *Yersinia pestis* [1], [2]. Nowadays, a number of other bacterial, viral and parasitic pathogens have been associated with rats, such as *Leptospira* spp., Shiga toxin producing *E. coli*, *Campylobacter* spp., *Salmonella* spp., or Hantaviruses [1], [3], [4], [5], [6]. There are numerous ways, by which rodent-borne pathogens may infect human and animal hosts. Inhalation of aerosols and consumption of contaminated food are considered the main pathways, while also direct contact, e.g. by bites, or infections via vectors might occur. Even surface water contaminated with droppings and urine from infected rats in recreational areas has been identified as possible infection source [7]. In addition, specific ecological and behavioral characteristics, e.g. a concentration of Brown rats into high-density populations along with their cohabitation with humans, may further promote the spread of zoonotic pathogens [7].

Another aspect of Brown ratś synanthropism is their inhabitation of areas near anthropogenically created food sources, such as garbage or sewage systems, also providing harborage [8], [9]. Although it is well known that rats live in certain parts of the sewage system [10], even continuous baiting programs have failed to eliminate Brown rat populations [11]. Brown rats from rural areas can roam as far as 260–2000 m within a day, while observational studies in city environments identified smaller activity areas of 25–150 m for rats in urban areas [10]. Nevertheless, urban Brown rats also appear to be able to build an epidemiological bridge between the sewage system and populated urban environments, as social factors, such as aggression in case of overpopulation of rats [12] or large disturbances in their environment can force populations to travel long distances also [13]. This can lead to large population fluctuations and the transmission of pathogens hosted by rats into new areas [14].

Although a natural fear of wild rats as putative carriers of infectious agents is largely embedded in our culture [15], there are hardly any scientific data regarding actual population trends. Estimations about the number of animals are scant or not available at all, like is also the case for our study site, Berlin. For other comparable urban areas, the total number of Brown rats seems to have been on a continuous high level over the last 50 years, as it has been reported for Baltimore (USA) [7]. But in recent years, there have also been reports on increased levels of infestation of urban areas in Great Britain [16]. At the same time, there is evidence of substantial under-reporting of rat infestations [10]. Furthermore, a deteriorating integrity of sewage infrastructures combined with less sewer baiting programs [10] may have intensified the occasion of direct and indirect contact between rat and humans in an urban environment. On a global level, climate change and changing human settlement patterns like the ongoing urbanization trend could lead to increased problems with rat-borne pathogens as the distribution of rodent species and pathogens linked to these species could be influenced [10].

Rats are natural hosts of *Escherichia* (*E*.) *coli,* a commensal ubiquitous bacterium colonizing the gut of mammals and birds [17]. Here, from a zoonotic perspective, intestinal pathogenic subtypes of *E. coli* (InPEC), including Shiga toxin producing *E. coli* (STEC), enterohemorrhagic *E. coli* (EHEC), enteroaggregative *E. coli* (EaggEC), and extraintestinal pathogenic *E. coli* (ExPEC) are of major concern. Recent studies on the occurrence of putatively zoonotic *E. coli* in rats were largely focused on STEC and on the epidemiologic relevance that rats, living on or in close proximity to cattle farms, might play in the distribution of EHEC O157 isolates [3], [5], [18]. Yet barely anything is known about the role of rats as carriers of ExPEC, which have received large attention recent years as they often express a multiresistance phenotype. In particular Extended-spectrum beta-lactamase (ESBL)-producing ExPEC strains account for serious problems in the treatment of infectious diseases in humans and animals as these enzymes confer resistance to nearly all beta-lactam antimicrobial drugs, including third-generation cephalosporins [19], [20], [21], [22], [23]. Although ESBL strains have been observed among all phylogenetic groups of *E. coli* including “extraintestinal pathogenic groups” B2 and D, still a larger proportion belongs to phylogenetic lineages and multilocus sequence types (STs) that are composed of opportunistic pathogens and commensals, lacking an extensive set of virulence-associated genes and causing infections primarily in immuno-compromised hosts [19], [21]. A well-known exception is the worldwide emerging clonal group O25b:H4-B2-ST131-CTX-M-15 which has been implicated in a wide range of severe hospital- and community-acquired extraintestinal infections in humans and animals [24]. The recent finding of an ESBL-producing *E. coli* strain belonging to this pandemic group in the feces of an urban rat from Berlin [22] prompted us to screen urban rats also for other multiresistant *E. coli*, the presence of genes associated with extraintestinal pathogenic and Shiga toxin producing strains, and their phylogenetic relatedness to human and animal clinical strains, determined by multi locus sequence typing (MLST). As the simple possession of virulence associated genes does not necessarily translate to *in vivo* pathogenicity, we chose one exemplary isolate to assess its pathogenicity in a chicken infection model. The strain was selected as it harbored a frequently encountered ESBL type (CTX-M-9) and represented a prominent and highly invasive ExPEC-lineage (ST95), which so far has been particularly associated with pathogenic human and avian strains and only scarcely expressed a multiresistance phenotype. The data obtained here might help to gain further insight into the role of synanthropic rodents as carriers, reservoir and even disseminators of *E. coli* that combine multiresistance and extraintestinal virulence.

## Materials and Methods

### Ethics Statement

All animal experiments were approved by the “Landesamt fuer Gesundheit und Soziales” (Reg. 0220/06) and chickens were killed according to animal welfare norms.

### Bacterial Strains

Fecal samples of 87 urban brown rats (*Rattus norvegicus)* were collected on 53 different sampling locations all over Berlin (Germany) from 2008–2009. Rats were captured and euthanized by pest control technicians during pest control (n = 40), or swabs were taken directly at the place of capturing and transferred into conservation medium (Mast Diagnostics, Reinfeld, Germany) (n = 47). After overnight cultivation on ChromeOrientation® agar (Mast Diagnostics) at 37°C, at least three *E. coli* isolates were obtained from each fecal sample. Classical biochemical methods were used to determine the bacterial species [25]. Copy clones recovered from individual animals (copy clones among different individuals were not detected), were excluded by randomly amplified polymorphic DNA (RAPD)-PCR, performed as recently described [26].

### Determination of Phenotypic Resistance and Pre-selection of Strains

Preliminary screening for antimicrobial resistance was done by agar dilution test with six different antimicrobial substances as recently described [27]. Here, freshly prepared Mueller–Hinton-agarose plates containing estimated breakpoint concentrations of ampicillin (≥32 µg/ml), streptomycin (≥64 µg/ml), spectinomycin (≥128 µg/ml), chloramphenicol (≥32 µg/ml), gentamicin (≥16 µg/ml) and tetracycline (≥16 µg/ml) were used. Isolates displaying phenotypic resistance for at least one antimicrobial class were additionally tested by Agar broth microdilution method (Micronaut breakpoint plate “Kleintier”, Genzyme Diagnostics, Rüsselsheim, Germany) against seventeen antimicrobials including beta-lactams as well as non-beta-lactams like aminoglycosides, tetracyclines, sulfonamides, chloramphenicol and fluoroquinolones according to the standards given by the CLSI guideline [28]. Phenotypic screening for ESBL production was performed using the confirmatory test with cefotaxime and ceftazidime alone or in combination with clavulanic acid according to the method recommended in the CLSI document M31-A3 [28].

### Determination of Antimicrobial Resistance Genes

Multiresistant *E. coli* isolates were screened for the presence of antimicrobial resistance genes, such as *tet*(A-D), *sul1*, *sul2*, *sul3*, *strA*, *strB*, *aadA1-like*, *aac(3)-IV*, *bla*_TEM-1-like_, *bla*_SHV_ and *bla*_CTX-M_ using standard PCR methods and sequencing of the PCR products if necessary. The presence of plasmid-mediated quinolone resistance gene variant *aac(6′)-Ib-cr* and the *qnrA*, *qnrB*, and *qnrS* genes as well as of mutations in *gyrA* and *parC* genes were determined by PCR and, if indicated by sequence or restriction analysis [29], [30], [31], [32], [33], [34], [35], [36], [37].

### Characterization of ESBL Producing Isolates

Self-transferability of plasmids was tested by mating experiments using azid-resistant recipient *E. coli* strain J^53^ as previously described [37]. Further characterization was performed by southern blotting, PCR-based replicon typing and pulsed-field gel electrophoresis (PFGE) using a CHEF DRIII System (BioRad, Munich, Germany) for comparative analysis with clinical isolates [37].

### Multilocus Sequence Typing and Phylogenetic Grouping

Multilocus sequence typing (MLST) was carried out for the multiresistant *E. coli* strains according to the scheme developed by Wirth et al. (2006) [17]. Gene amplification and sequencing was done by using primers specified at the *E. coli* MLST web site (http://mlst.ucc.ie/mlst/mlst/dbs/Ecoli). Sequences were analyzed by the software package RidomSeqSphere (http://www.ridom.de/) and STs were either computed automatically or newly assigned in case novel STs have been identified. *E. coli* phylogenetic groups were determined by Structure analysis based on the concatenated sequences of the seven housekeeping genes (http://pritch.bsd.uchicago.edu/structure).

### Virulence Gene Typing

**Figure 1:**
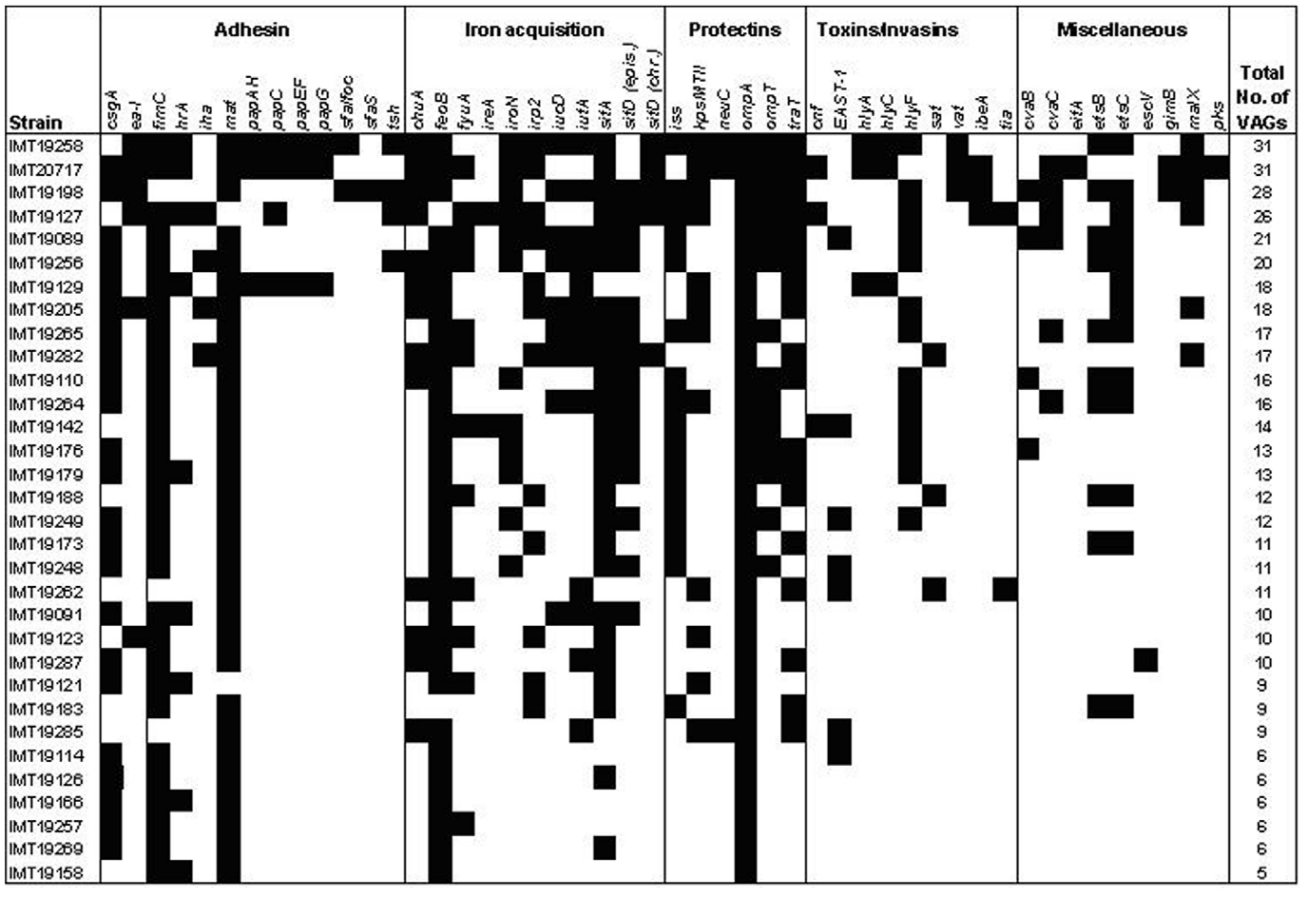
Distribution of virulence-associated genes among 32 multiresistant rat *E. coli* isolates. The following genes showed no positive results and are not presented in the figure: *bfp, bmaE, eae, eitC, focG, gafD, nfaE, pic, pks, puvA, stx1* and *stx2*.

Multiresistant *E. coli* isolates were examined for the presence of 59 virulence-associated genes (VAGs) linked with extraintestinal pathogenic and Shiga toxin producing *E. coli* by multiplex and single PCRs as described previously [37]. VAGs determined encode factors within the categories of toxins, adhesins, iron aquisition systems, protectins and others (detailed information is given in Fig. 1).

### Chicken Infection Model

Based on phenotypic and genotypic resistance pattern, MLST and virulence gene typing, one exemplary rat isolate (IMT20717; O18:NM:K1; ST95; ST complex 95), resembling a highly virulent ExPEC genotype and serogroup and additionally expressing a CTX-M-9-type beta lactamase, was selected to assess it pathogenic potential *in vivo.*

A chicken infection model [38], which has already been shown to be appropriate for testing non-avian ExPEC strains as well [39] was used. Four groups of six chickens each were infected intra-tracheally with 10^9^ colony-forming units (CFU) of the test strain and two control strains, one low pathogenic avian fecal strain (IMT12226; O77:H18; ST1165, STC144) as negative control and archetypical *E. coli* strain RS218 (O18:H7:K1; ST95; STC95), isolated from a case of meningitis in a baby, as positive control strain [40]. This non-ESBL producing strain was included in order to directly compare a clinical strain of the same phylogenetic background and identical O- and K-antigens with the multiresistant urban rat isolate.

## Results and Discussion

### High Isolation Rates of *E. coli* from Rats

*E. coli* isolates were recovered from 77.0% (n = 67) of the 87 rats (*R. norvegicus*). Isolation from fresh fecal swabs (95%) seemed more preferable than that from swabs taken to the laboratory in conservation media (70%). Overall, the isolation rate is beyond what has been described for other rodent fecal samples so far [41], whereas it is similar to what has been found in a study including wild *R. rattus* in Africa [42]. After picking at least three colonies per animal, a total of 238 *E. coli* isolates were obtained. RAPD-PCR analysis (data not shown) was further used to exclude isolates with identical band profiles, which were only observed in individual animals but not in-between rats. Finally, 211 non-duplicate strains were included in further experiments and the reduction by a number of only 27 copy strains indicated a quite high diversity amongst rat *E. coli* isolates.

### Frequent Occurrence of Multiresistant *E. coli* Isolates in Rats

Several rat *E. coli* isolates showed phenotypic resistance to ampicillin (15%), cephalothin (5%), as well as to fluoroquinolones like enrofloxacin (7%), difloxacin (9%) and marbofloxacin (7%), the two aminoglycosides gentamicin/kanamycin (both 5%), tetracycline (16%), sulfamethoxazole-trimethoprim (9.4%), and chloramphenicol (10%). A total of 55 of all 211 isolates (26%) exhibited resistant phenotypes in the agar dilution test against at least one antimicrobial class. According to MIC data, 32 of these isolates showed resistance to three or more classes of antimicrobials (Table 1). Thus, following the definition given by Schwarz et al. (2010) [43], overall 13.6% (n = 32) of *E. coli* strains isolated from urban rats should be regarded multiresistant. Of these, two (IMT19205 and IMT20717) showed a positive confirmatory test for the production of ESBLs. One of these isolates (IMT19205) belonged to the pandemic clonal group B2-ST131-O25b:H4 and was included in a previous publication [22]. MIC testing of all 32 isolates revealed high rates of resistance to beta-lactams like ampicillin (87.5%), oxacillin (96.9%), cephalothin (31.3%), as well as to fluoroquinolones like enrofloxacin (43.8%), difloxacin (50%) and marbofloxacin (43.8%), the two aminoglycosides gentamicin/kanamycin (both 34.4%), tetracycline (84.4%), sulfamethoxazole-trimethoprim (59.4%), and chloramphenicol (65.6%). The most abundant pattern observed was combined resistance to ampicillin, tetracycline and the fluoroquinolones (Table 1). Screening for antimicrobial resistance determinants nearly always reflected the phenotypic resistance situation. Most or all strains harboured *bla*_TEM-1-like_ (87.5%), *sul1/sul2* (75%) and *strA/B* genes (100%), whereas other non-beta-lactam resistance genes, such as *aadA* (34.4%), *tet*(A–D) (25%), *aac(3)IV* (3.1%), *aac(6′)-Ib-cr* (6.3%), and *qnrB1* (3.1%) were present in lower frequencies (Table 1).

**Table 1:** Results of genotypic and phenotypic characterisation of 32 multiresistant rat *E. coli* isolates.

Strain No.	PG	ST	STC	No. VAGs	Phenotypic resistance for antimicrobial substances [no. of classes with phenotypic resistance]	Mutated amino acids encoded in*				Antimicrobial resistance genes/gene variants
						*gyrA*		*parC*		
						Ser83	Asp87	Ser80	Glu84	
IMT19258	B2	127	127	31	*AMP-OXA-ENR-MAF-DIF-ORB-GEN-KAN-TET-TS* [5]	WT	WT	WT	WT	*bla*_TEM-1-like_, *sul1, sul2, strA, strB*
IMT20717	B2	95	95	31	*CEF-AMP-OXA-GEN-KAN-TS* [3]					*bla*_CTX-M-9_, *bla*_TEM-1-like_, *sul2, strA, strB, aac(6′)-Ib-cr, aadA*
IMT19198	B2	1851	none	28	*AMP-OXA-TET-TS* [3]					*bla*_TEM-1-like_, *sul1, strA, strB*
IMT19127	B2	428	none	26	*AMP-OXA-CTN-TET-TS* [3]					*bla*_TEM-1-like_, *tet*(B), *sul2, strA, strB, aadA*
IMT19089	B1	88	23	21	*AMP-OXA-CTN-MAF-TET* [3]	WT	WT	WT	WT	*bla*_TEM-1-like_, *sul1, sul2,strA, strB, aadA*
IMT19256	ABD	57	350	20	*AMP-OXA-ENR-MAF-DIF-ORB-TS* [3]	WT	WT	WT	WT	*bla*_TEM-1-like_, *sul2, strA, strB, aadA, qnrB1*
IMT19129	D	38	38	18	*AMP-OXA-CTN-GEN-TET-CMP* [4]					*bla*_TEM-1-like_, *strA, strB*
IMT19205	B2	131	131	18	*CEF-AMP-OXA-ENR-MAF-GEN-TET* [4]	Leu	Asn	Ile	Val	*bla*_CTX-M-9_,*bla*_TEM-1-like_, *tet*(A) *sul2, strA, strB, aac(6′)-Ib-cr, aac(3)IV*
IMT19265	AxB1	93	168	17	*AMP-OXA-ENR-MAF-DIF-ORB-TS* [3]	WT	WT	WT	WT	*bla*_TEM-1-like_, *bla*_OXA-1_, *sul1,strA, strB, aadA*
IMT19282	B2	2381	none	17	*AMP-OXA-KAN-TET-CMP* [4]					*bla*_TEM-1-like_, *tet*(D), *strA, strB*
IMT19110	D	1011	none	16	*AMP-OXA-ENR-MAF-DIF-ORB-GEN-TET-TS-CMP* [6]	Leu	Asn	Ile	WT	*bla*_TEM-1-like_, *sul1, strA, strB*
IMT19264	AxB1	93	168	16	*AMP-OXA-TET-TS* [3]					*bla*_TEM-1-like_, *bla*_OXA-1_, *sul1, strA, strB, aadA*
IMT19142	ABD	1850	none	14	*AMP-OXA-CTN-DIF-TET-CMP* [4]	Leu	WT	WT	WT	*bla*_TEM-1-like_, *sul1, sul2, strA, aadA*
IMT19176	ABD	641	86	13	*AMP-OXA-CTN-ENR-DIF-TET-TS-CMP* [5]	Leu	WT	WT	WT	*bla*_TEM-1-like_, *sul1, sul2, strA, strB, aadA*
IMT19179	A	1286	none	13	*AMP-OXA-KAN-TET-CMP* [4]					*bla*_TEM-1-like_, *sul1, strA, strB*
IMT19188	B1	1049	none	12	*AMP-OXA-CTN-TET-CMP* [3]					*bla*_TEM-1-like_, *sul1, strA, strB*
IMT19249	ABD	1850	none	12	*AMP-OXA-ENR-DIF-TET-CMP* [4]	Leu	WT	WT	WT	*bla*_TEM-1-like_, *tet*(D), *strA, strB, aadA*
IMT19173	B1	1049	none	11	*AMP-OXA-TET-CMP* [3]					*bla*_TEM-1-like_, *sul1, strA, strB*
IMT19248	ABD	1850	none	11	*AMP-AMC-OXA-DIF-TET-TS-CMP* [5]	Leu	Asn	WT	WT	*bla*_TEM-1-like_, *sul1, strA, strB*
IMT19262	D	501	none	11	*OXA-ENR-MAF-DIF-ORB-TS-CMP* [4]	WT	WT	Ile	WT	*bla*_TEM-1-like_, *sul1, sul2, strA, strB*
IMT19091	A	10	10	10	*AMP-OXA-CTN-ENR-MAF-DIF-ORB-GEN-TET-TS-CMP* [6]	Leu	Asn	Ile	WT	*bla*_TEM-1-like_, *strA, strB*
IMT19123	D	70	none	10	*OXA-TET-TS* [3]					*strA, strB*
IMT19287	AxB1	1849	none	10	*MAF-DIF-GEN-CMP* [3]	WT	WT	WT	WT	*bla*_TEM-1-like_, *tet*(D), *sul1, strA*
IMT19121	ABD	453	86	9	*AMP-OXA-CTN-TET-CMP* [3]					*strA, strB*
IMT19183	B1	1049	none	9	*AMP-OXA-TET-CMP* [3]					*bla*_TEM-1-like_, *sul1, strA, strB, aadA*
IMT19285	D	501	none	9	*AMP-OXA-ENR-MAF-DIF-ORB-GEN-KAN-TET-TS-CMP* [6]	Leu	Asn	Ile	WT	*bla*_TEM-1-like_, *sul1, strA, strB*
IMT19114	B2	1444	none	6	*AMP-OXA-TET-CMP* [3]					*bla*_TEM-1-like_, *sul1, strA, strB*
IMT19126	ABD	641	86	6	*AMP-OXA-TET-CMP* [3]					*bla*_TEM-1-like_, *tet*(D), *strA, strB*
IMT19166	AxB1	224	none	6	*AMP-OXA-ENR-MAF-DIF-ORB-GEN-KAN-TET-TS-CMP* [6]	Leu	Asn	Ile	WT	*bla*_TEM-1-like_, *sul1, strA, strB, aadA*
IMT19257	B1	2380	none	6	*OXA-TET-CMP* [3]					*sul2, strA, strB*
IMT19269	AxB1	1849	none	6	*AMP-OXA-ENR-MAF-DIF-ORB-TET-TS* [4]	Leu	WT	WT	WT	*bla*_TEM-1-like_, *tet*(D), *sul1, strA, strB*
IMT19158	B1	2976	none	5	*AMP-OXA-ENR-MAF-DIF-ORB-GEN-KAN-TET-TS-CMP* [6]	Leu	Asn	Ile	WT	*bla*_TEM-1-like_, *sul1, strA, strB*

Footnote to Table 1, sorted by the number of virulence associated genes (VAGs, column 5).

AM = antimicrobial, PG = ancestral/phylogenetic group, ST = sequence type, STC = sequence type complex, VAGs = virulence associated genes, AMP = ampicillin, AMC = ampicillin/clavulanic acid, CEF = cefotaxim, CMP = chloramphenicol, CTN = cefotetan, DIF = difloxacin, ENR = enrofloxacin, GEN = gentamicin, KAN = kanamycin, MAF = marbofloxacin, ORB = orbifloxacin, OXA = oxacillin, TET = tetracycline, TS = sulfamethoxazole/trimethoprim.

* empty fields: not tested (fluoroquinolone sensitive strains).

In general, data on antimicrobial resistance in *E. coli* from wild rats are rather limited. Literak et al. (2009) identified 2.5% of African *R. rattus* isolates to be ESBL-producers [42]. An additional study reported high rates of multiresistant *E. coli* in rats *(R. norvegicus)* from a port in Greece [6]. Taking into account other synanthropic wildlife species as well, the rates of antimicrobial resistant *E. coli* detected in this study are higher than what has been found in raccoons (16% from urban environments) [44] or small mammals (15% in residential areas) [45]. The higher rates obtained from urban rats could be explained by the assumption that human activities including production of sewage are the most likely common source of *E. coli* transmission to urban wildlife. As only rats populate the sewage system directly, they have direct contact with human feces, whether from private households or clinics and might frequently take up multiresistant strains in this way. The recent finding of comparable antimicrobial resistance patterns in *E. coli* isolates from rats and humans agrees with this [46], [47]. Also, compared to rodents from rural areas in Central Europe, the rates of multiresistant *E. coli* from urban rats seem to be higher (13.6% vs. approx. 2%) [22], [48]. One logical conclusion could be that rats might serve as surrogate marker for the spread of antimicrobial resistance in urban areas. Above all, however, their potential to disseminate multiresistant microorganisms in highly populated areas should not be obscured, especially since there is almost no doubt about their ability to spread zoonotic pathogens among humans and animals [3], [4], [5], [49].

### High Diversity of Sequence Types (STs) Among Multiresistant Rat Isolates Including STs Associated with Extraintestinal Pathogenicity in Humans

Overall we determined a total of 24 different STs among the 32 multiresistant isolates out of which seven were assigned to ancestral group B2 (ST95, ST131, ST127, ST428, ST1444, ST1851, and ST2381), four to group D (ST38, ST70, 2×ST501), three to group B1 (ST88, ST2380, and ST2976), and two to group A (ST10 and ST1286) (Tab.1). Another eleven and five STs belonged to hybrid groups ABD (ST57, ST453, ST1011, 2×ST6413×ST1049, and 3×ST1850), and AxB1 (2×ST93, ST224, and 2×ST1849), respectively, which are supposed to represent highly recombining groups that have gained genetic material from different ancestral groups in the past [17]. More than one third (34.5%) of multiresistant strains were allocated to the ExPEC-linked phylogenetic groups B2 and D. This high rate is quite surprising as the B2 group generally represents the minority of ESBL-producing *E. coli,* when compared with the remaining groups, which more frequently harbor antimicrobial resistances [21], [50], [51]. Nevertheless, the ST131-O25b:H4 pandemic clonal group also belongs to group B2 and its success might just hallmark an ongoing development where B2 strains are becoming increasingly multiresistant. Interestingly, ESBL-producing *E. coli* isolate IMT20717 was a B2 strain affiliated to ST95, which currently represents one of the key ExPEC lineages in humans [52], accounting for about 10% of human ExPEC strains deposited into the database (last access: 04.10.2012; http://mlst.ucc.ie/mlst/mlst/dbs/Ecoli/). Human ST95 strains extensively share virulence features with ST95 strains isolated from systemic infections in poultry, and so far this phylogenetic lineage seemed to be almost exclusively linked with these two species [17], [39], [52], [53]. Although we neither have evidence nor epidemiologic support, due to the high species linkage of ST95 strains the rat isolate IMT20717 might have its primary source rather in a human or bird individual than in the rat itself, supporting putatively ongoing transmission cycles.

### Frequent Occurrence of Virulence Genes Associated with Extraintestinal Pathogenicity Among Multiresistant Rat Isolates

None of the multiresistant *E. coli* strains harboured Shiga toxin genes 1 and 2, nor did we detect genes encoding for adherence factors intimin (*eae*) and bundle forming pili (*bfp*), among others indicating the absence of STEC among the multiresistant rat strains. So far, Shiga toxin producing *E. coli*, including EHEC O157, have only been identified in samples from rats living in close proximity to cattle farms or with access to feedlot-cattle water tanks [5], [18], [54]. This epidemiologic link was definitely not given in case of our sample material and our results were therefore quite reasonable. In contrast, we frequently detected a number of ExPEC-related genes, as shown in Fig. 1. Overall 17.2% of all multiresistant rat strains harboured at least twenty VAGs (max. of 31), most of these belonging to the B2 group and thus presenting typical ExPEC strains.

Nearly all isolates harboured bacterial adhesin genes encoding Type 1 (*fimC*)- and Curli fimbriae (*csgA*). Also the presence of typical ExPEC-related adhesins, such as the heat-resistant agglutinin [*hrA* (28.1%)], iron-regulated hemagglutinin [*iha* (12.5%)], P-fimbriae [*pap* operon genes (9.4%–12.5%)], S-fimbriae [*sfa/foc* (6.3%)], or a recently described ExPEC adhesin [*ea/I* (18.8%) hinted towards the affiliation of a number of rat strains to the group of ExPEC strains. Iron acquisition genes, such as *chuA* (43.8%), *fyuA*, *iroN*, *irp2* (all 37.5%), *iucD* (28.1%), *iutA* (40.6%), *sitA* (78.1%), and *sitD*_episomal_ (46.9%), which are known to confer fitness advantage and also invasive properties towards *E. coli* residing in the gut or bladder of their host, under certain circumstances being capable of causing infections at various extraintestinal sites [55], [56], [57], were also frequently detected. The finding of protectin genes like increased serum resistance gene *iss* (53.1%), and invasion-associated K1-capsule encoding gene *neuC* (9.4%), as well as of plasmid-located transfer [*traT* (56.3%)] and outer membrane genes [*ompT* (43.8%)], all of which are highly associated with the virulence of human and avian ExPEC strains [52], [58] substantiates our belief, that rats could frequently be asymptomatically colonized by ExPEC-like strains and may thus serve as a permanent source of zoonotic *E. coli*. The pathogenic nature of a number of the strains isolated in the present study is further supported by the detection of toxin genes, such as the cytonecrotizing factor *cnf* (12.5%), secreted autotransporter toxin *sat* (9.4%), vacuolating autotransporter toxin *vat* (9.4%), and haemolysin operon genes *hlyA* and *hlyC* (9.4%), which are particularly characteristic for uropathogenic *E. coli* [56]. Apart from the K1-capsule, which is one of the main features of highly invasive ExPEC strains, exemplified by a subgroup of avian pathogenic *E. coli* (APEC) as well as by *E. coli* strains implicated in new-born meningitis (NMEC), we also found other invasion-related factors among the rat strains, including *ibeA* (9.4%), which has a crucial role in the bacterial translocation of the blood brain barrier epithelium and *in vivo* pathogenicity, as previously shown in a rat meningitis and a chicken infection model [59], [60].

Consistent with our results, recent publications attributed the successful colonization of the healthy gut of humans, dogs, swine, and poultry also to the presence of ExPEC-typical VAGs [53], [61], [62], [63] The frequent finding of multiresistant ExPEC-like strains among rat samples, however, contradicts the paradigm about an ultimate loss of bacterial fitness due to the maintenance of antibiotic resistance in combination with high levels of virulence [64]. This combination is considered one of the major drivers for the international spread of ESBL clone O25b:H4-B2-ST131, while there are also studies pointing out that this might be only one side of the coin [21], [65]. If virulence would be that decisive for the emergence of antimicrobial resistant and highly virulent ExPEC strains, one would expect other clonal groups, such as the B2-ST95 lineage, which accumulates highly invasive, mostly human and avian strains [17], [39], [52], [53], to acquire a multiresistance phenotype, by that amplifying its threat to human and animal health. Though, as discussed earlier, so far only a marginal proportion (4%) of all ST-complex 95 strains deposited on the web-hosted database (http://mlst.ucc.ie/mlst/mlst/dbs/Ecoli/) or reported in several publications harbors ESBL genes or simply a multiresistant phenotype [21]. The more intriguing it was that we identified an ST95 ESBL-producing strain (IMT20717; CTX-M-9) among the rat isolates, which remarkably showed multiresistance, frequent possession of virulence genes (n = 31) in a B2 phylogenetic background, and a serogroup (O18:NM:K1) typical of highly invasive ExPEC strains (Fig. 1; Table 1). In that way it very much resembles isolates causing urosepsis and new born meningitis in humans, and septicemia in chickens. Due to its observed lifestyle in the rat, namely asymptomatically colonizing the gut, it was reasonable to deduce the strainś extraintestinal pathogenicity not simply from its phylogenetic background and the possession of several VAGs, but also experimentally in an *in vivo* model. We made use of chicken experiments as this has been shown a proper model for determining the pathogenicity of ExPEC strains, in particular of ST95 strains, which are highly linked to chickens as one of their natural hosts [39].

### Paradigmatic Combination of Multiresistance and Extraintestinal Pathogenicity in Urban Rat ST95-CTX-M-9-producing Strain IMT20717

IMT20717 displayed a positive confirmatory test for the production of ESBL. Apart from a *bla*_CTX-M-9_ gene, this strain also harboured resistance genes *bla*_TEM-1_, *sul2*, *strA*, *strB, aac(6′)-Ib-cr* and *aadA.* All these genes, except for *sul2*, were located on a self-transferable, approximately 50 kb plasmid of the N/FIC replicon type.

**Figure 2:**
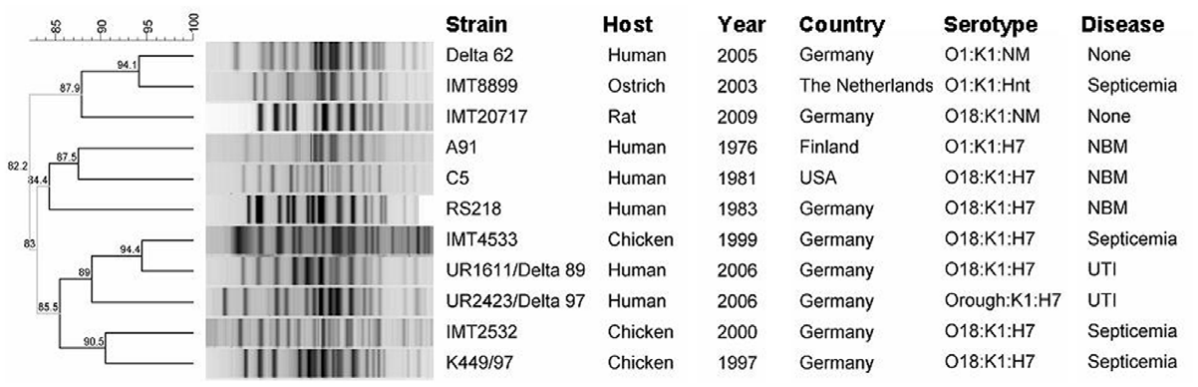
Dendrogram of ST95-ESBL rat strain IMT20717 with *E. coli* ST95-K1 strains. The clonal relationship shown is based on *XbaI*-generated PFGE profiles. NM = non motile (H antigen negative or not expressed); NBM = newborn meningitis; UTI = urinary tract infection, optimization 1.0%, position tolerance 1.5%.

Serotyping characterized IMT20717 as O18:NM:K1. Thereby the strain expressed a combination of an O-antigen and a capsule type which is highly linked with a clonal group of *E. coli* strains frequently involved in invasive infections in humans [19], [39], [58], [59], [66]. Particular attention has been drawn to *E. coli* O18:K1 NMEC strains causing meningitis in babies shortly after delivery. In addition, this serogroup is also frequent among avian pathogenic *E. coli* (APEC). Here, it causes often fatal septicemia and is responsible for great losses in poultry breeding [39], [52], [66]. Macrorestriction analysis and subsequent PFGE revealed a high genetic similarity (Dice similarity ≥ 82.2%) between the rat B2-ST95-O18:NM:K1-CTX-M-9 isolate and clinical ST95 strains of different ExPEC pathovars, and an additional fecal strain from the gut of a healthy human, all affiliated to this globally distributed lineage (Fig. 2). This similarity strongly resembles what is already well known, in that, the healthy human gut serves as a reservoir for these pathovars [61]. The detection of a pathogenic strain linked to human clinical environments points towards a possible transmission pathway through clinic waste into the urban sewage system.

**Figure 3:**
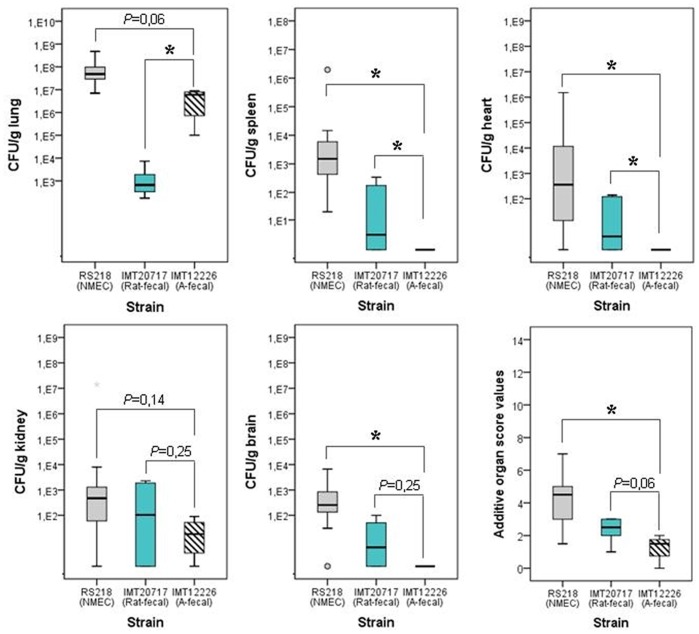
Results of the ST95-ST95 ESBL rat strain IMT20717 in the chicken infection model. Ability of B2-ST95-O18:NM:K1-CTX-M-9 urban rat strain IMT20717 to colonize the lungs, disseminate into internal organs and penetrate the blood brain barrier 24 h post intra-tracheal infection (10^9^ CFU) of a group of six 5-weeks old SPF White Leghorn chickens. Non-ESBL-producing NMEC strain RS218 (B2-ST95-O18:H7:K1) and avian fecal strain IMT12226 (ST1165-O77:H18), known invasive and low pathogenic strains, were used as controls.

In the *in vivo* infection model IMT20717 revealed a lower bacterial recovery rate from chicken organs than the clinical NMEC type strain RS218 (Fig. 3), which was included for comparative purposes. Nevertheless the strain could be isolated from all internal organs in significantly higher numbers than the low pathogenic avian control strain IMT12226. Particularly its re-isolation from the brain is of high indicative value for its invasive potential (Fig. 3) as it suggests that the strain is able to penetrate the blood brain barrier. In view of this, the finding of an ST95 strain in the gut of a rat could add rats to the list of potential hosts for highly extraintestinal pathogenic *E. coli* as well. What is even more disturbing is the fact that this strain carried an ESBL-encoding plasmid. Such a combination, namely ESBL-ST95- *E. coli*, has only rarely been observed in human clinical samples so far [21] and its detection in wild rat is the first description of such a superbug in an animal.

### Conclusions

The urban rats examined in this study frequently carried multiresistant *E. coli* strains showing high levels of resistance to critically important antimicrobials like fluoroquinolones and ß-lactams. As the WHO classified urban rats as a significant public health threat [2] the data reported here might have yet unpredictable consequences in the future. In particular, the finding of an ESBL-producing ExPEC strain belonging to one of the most virulent ExPEC lineages (ST95) might signify a new development in the field of antimicrobial resistance, in that ESBL plasmids step by step could find their way into highly virulent *E. coli* populations. There is still no final clue for the recent dominance of the pandemic B2-ST131-O25b:H4-CTX-M-15 clonal group. Several non-resistance-related attributes like bacterial fitness, virulence patterns or insertional modifications in fimbrial genes have been discussed as putative causes [65], [67], [68]. Taking into account the virulence potential of ST95, which is believed to be comparably high to that of ST131, it remains unclear why ST95 is far from being as broadly distributed as ESBL-producing ST131. However, the urban rat-derived B2-ST95-O18:NM:K1-CTX-M-9 strain possesses a number of genetic markers whose products confer adhesive, toxic and invasive properties and thus meets all requirements for a successful commensal and extraintestinal pathogenic life style. Future monitoring of clinical and environmental ESBL *E. coli* isolates should therefore clarify whether the detection of this ST95-ESBL strain from a rat simply presents an accidental finding of a minor important ESBL clone in a single animal, or whether it points towards a successful spread of ST95-ESBL outside the clinics as well. In any case, this strain hallmarks the main finding of this study: the mere occurrence of *E. coli* strains in urban rats that are multiresistant & virulent is an alarming observation, as infections with such strains could lead to severe clinical outcomes, leaving only limited treatment options.
